# Are the processes of DNA replication and DNA repair reading a common structural chromatin unit?

**DOI:** 10.1080/19491034.2020.1744415

**Published:** 2020-04-10

**Authors:** Stefania Mamberti, M. Cristina Cardoso

**Affiliations:** Cell Biology and Epigenetics, Department of Biology, Technische Universität Darmstadt, Darmstadt, Germany

**Keywords:** Chromatin structure, chromatin function, DNA structure, DNA replication, DNA repair, high resolution microscopy, polymer modeling

## Abstract

Decades of investigation on genomic DNA have brought us deeper insights into its organization within the nucleus and its metabolic mechanisms. This was fueled by the parallel development of experimental techniques and has stimulated model building to simulate genome conformation in agreement with the experimental data. Here, we will discuss our recent discoveries on the chromatin units of DNA replication and DNA damage response. We will highlight their remarkable structural similarities and how both revealed themselves as clusters of nanofocal structures each on the hundred thousand base pair size range corresponding well with chromatin loop sizes. We propose that the function of these two global genomic processes is determined by the loop level organization of chromatin structure with structure dictating function.

**Abbreviations:** 3D-SIM: 3D-structured illumination microscopy; 3C: chromosome conformation capture; DDR: DNA damage response; FISH: fluorescent in situ hybridization; Hi-C: high conformation capture; HiP-HoP: highly predictive heteromorphic polymer model; IOD: inter-origin distance; LAD: lamina associated domain; STED: stimulated emission depletion microscopy; STORM: stochastic optical reconstruction microscopy; SBS: strings and binders switch model; TAD: topologically associated domain

## Graphical Abstract

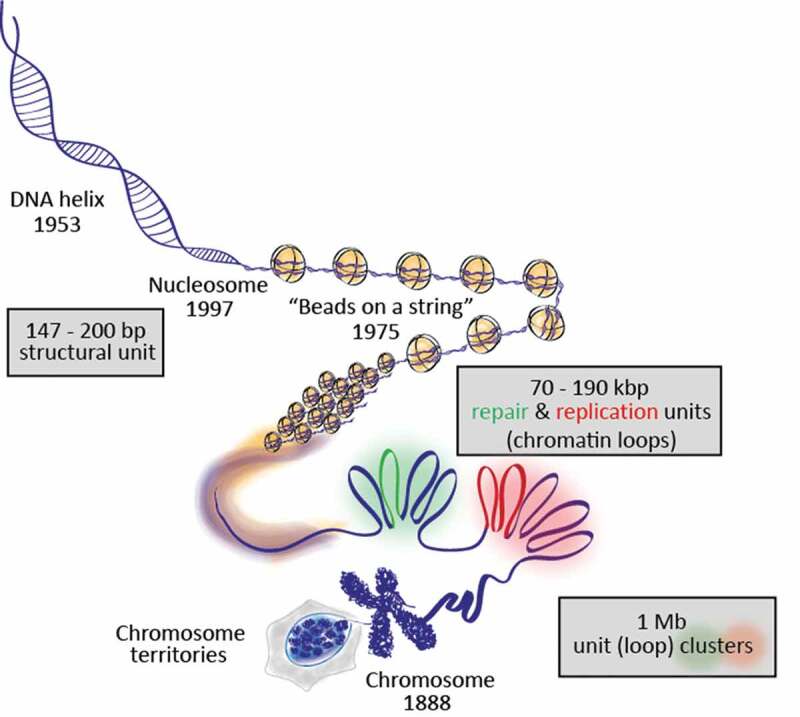

## Introduction

The biggest polymer in cells happens to be DNA and the least is known about its structure and how this relates to its function. In recent years, the 4D Nucleome program (https://www.4dnucleome.org) was founded exactly to tackle the issue of how the genome is folded in three-dimensions, how this dynamically changes in time (the fourth dimension) and which are the functional consequences of such folding [1,2]. It is also known for quite some time that genomic processes occur within discrete subnuclear sites. However, whether the structure of these sites is determined by or rather determines DNA metabolism remains to be elucidated.

Indeed, in the last decades, a variety of methods have been developed toward unraveling how DNA is organized within the nucleus. These include: light and electron microscopy-based approaches (e.g., [3–5]), DNA metabolism-based techniques such as the incorporation and detection of nucleosides’ analogues (reviewed in [6]), DNA halo visualization (e.g., [7]), fluorescent in situ hybridization (FISH) (e.g., [8]), chromosome conformation capture (3 C) based methods ([9], reviewed in [10]) and polymer modeling (reviewed in [11,12]).

Here, we present our recent discoveries on the structural organization of chromatin units of the global genomic processes of DNA replication and repair [13–15], in light of the interplay between genome structure and function.

## The chromatin organizing factors cohesin and CTCF

Increasing evidence has established the architectural proteins CTCF and the cohesin complex as major players in genome organization, as extensively reviewed before (see, e.g.,, [16–18]). Genome function and its structural organization have indeed coevolved during the branching process of the tree of life. While cohesin-like proteins are found already in prokaryotes [19,20], CTCF is conserved in most bilaterian metazoan and might have impacted the body patterning across Bilateria by forming the kernel of a gene regulatory network together with the *Hox* genes, through its role in chromatin domain formation [21,22].

CTCF is an eleven-zinc-finger DNA binding protein, which was initially discovered for its transcriptional regulation of the chicken *c-myc* gene [23,24]. This protein was shown to mediate the insulation of a chromatin loop by bringing together two distant DNA sites, after binding sequence-specific DNA sites in a convergent orientation [25].

Cohesin is a ring-shaped protein complex, which was primarily known to provide cohesion between two sister chromatids after DNA replication (reviewed in [16]). More recently, the complex has been proposed to load on DNA and to extrude a loop until being removed by the release factor Wapl or until encountering an obstacle such as CTCF, as stated in the loop extrusion model [25, reviewed in 26, see also below].

## Is DNA hierarchically folded into chromatin units of defined size?

**Figure 1.**
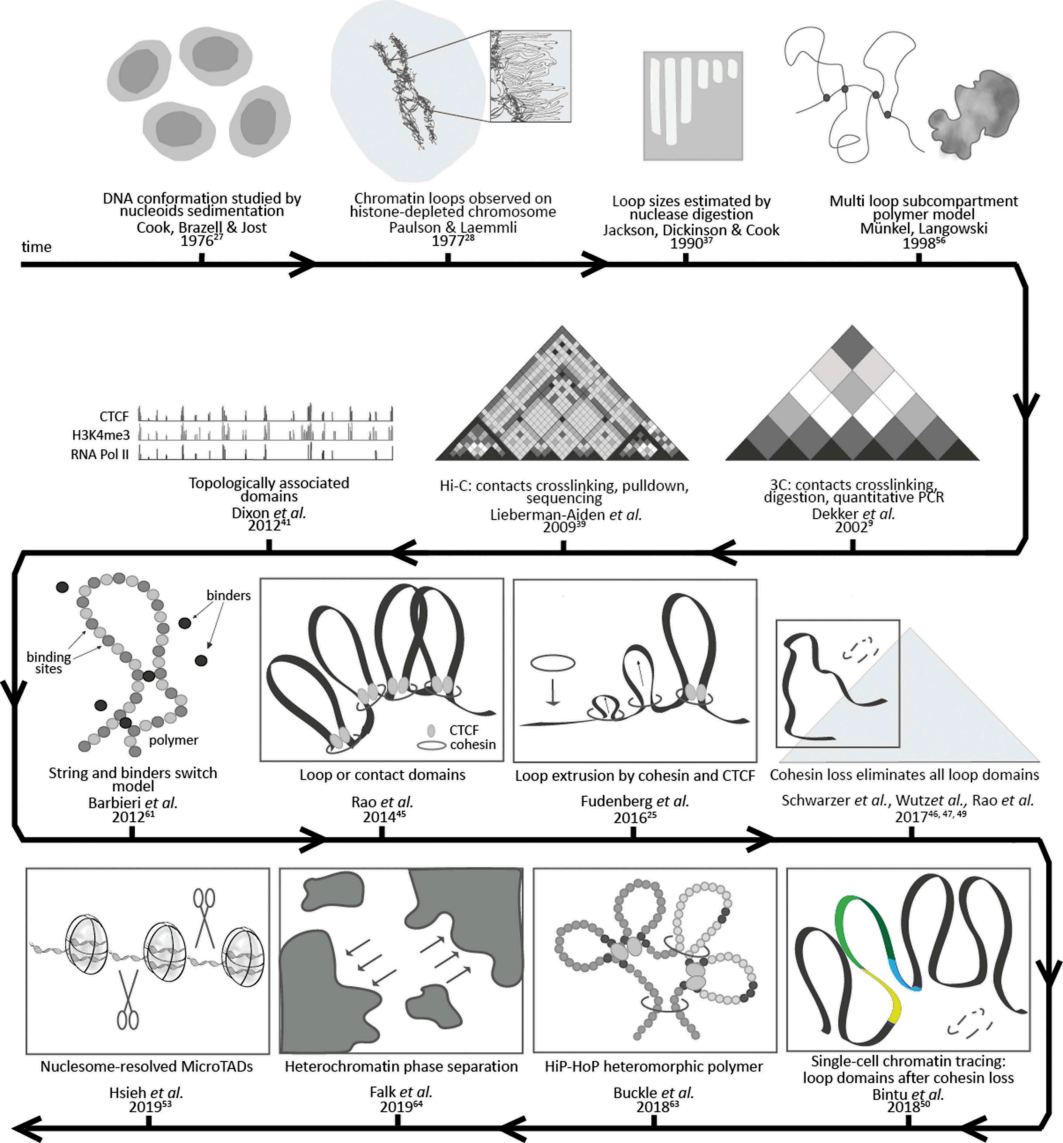
Timeline of measurements and modeling of chromatin structures.

A variety of studies investigating how genomic DNA is folded over multiple decades are listed in Table 1, the timeline Figure 1 and discussed below.

**Table 1.** Sizes of structural chromatin units measured with different methods.

	Reference	Year	Method	Nomenclature/Structure	Organism (cell line)	Median/ mean size	Size range
Structure	Paulson and Laemmli	1977	Histone-depleted metaphase chromosomes	Loop	Human (HeLa)	70 kb	30 – 90 kb
	Vogelstein, Pardoll & Coffey	1980	DNA Halo technique	Loop	Mouse (3T3)	90 kb	84 – 96 kb
	Buongiorno-Nardelli *et al.*	1982	Halo technique	Loop	Frog (X. laevis erythrocytes and kdiney cells)	90 kb	-
	Earnshaw and Laemmli	1983	Metaphase chromosome	Loop	Human (HeLa)	83 kb	± 29 kb
	Jackson, Dickinson and Cook	1990	Nuclease digestion and electrophoresis	Loop	Human (HeLa)	86 kb	5 – 200 kb (80–90 kb)
	Lieberman-Aiden *et al.*	2009	Hi-C	Megadomains	Human (GM06990)	-	5 Mb – 20 Mb
				A/B compartments		-	500 kb – 7 Mb
	Dixon *et al.*	2012	Hi-C	TADs	Mouse (mESCs)	880 kb	100 kb – 5 Mb
	Rao *et al.*	2014	Hi-C	Loop domains	Human and mouse cell lines	185 kb	40 kb – 3 Mb
	Gibcus *et al.*	2018	Hi-C combined with polymer simulation	Inner loops in prophase	Chicken (DT-40)	60 kb	-
				Inner loops in prometaphase		80 kb	-
				Nested outer loops in prometaphase		400 kb	-
	Hsieh *et al.*	2019	Micro-C	MicroTADs	Mouse (mESCs)	5.4 kb	1 – 32 kb

In 1976, Cook, Brazell & Jost propose the involvement of loops in the superhelical organization of the genome, meant as organization level above the double helix [27]. They prepared nucleoids from human HeLa cells with a lysis solution containing a nonionic detergent and variable concentrations of salt, up to a saturating level, thus depleting histones. Based on sedimentation ratios of these nucleoids through sucrose gradients containing the intercalating DNA dye ethidium bromide, they were able to deduce their DNA conformation. They did not observe any effect of the nonionic detergent and saturating concentration of salt (conditions that removed most chromatin proteins including histones) in the migration of the nucleoids through the gradient. Therefore, they concluded that additional constraints existed, which kept the superhelical organization of the DNA duplex intact.

The year after, 1977, Paulson and Laemmli used electron microscopy to study histone-depleted metaphase chromosomes, obtained by treating purified HeLa cells chromosomes with dextran sulfate and heparin [28]. They showed that most of the DNA existed in loops of at least 10–30 μm, appearing as a halo, held together by a scaffold of non-histone proteins, or core, shaped characteristically as a metaphase chromosome. Assuming that 1 μm of DNA would equal 3000 base pairs [29], they calculated a DNA content of 30–90 kb per loop. They proposed that their measurements might be underestimates, due to the fact some DNA may not have been completely unfolded and to the observation that a few loops were longer than 60 μm. However, they pointed out that similar loop sizes were observed in *E. coli* [30] and from sedimentation studies of eukaryotic interphase cell nuclei [31,32]. They also highlighted the fact that, in a separate study, they could demonstrate that the scaffold could be isolated as an entity independent of DNA, by treating the chromosomes with micrococcal nuclease before depleting them of histones [33,34], suggesting that non-histone proteins are responsible for the higher-order organization of eukaryotic chromatin.

In 1980, Vogelstein and colleagues first applied the DNA halo technique, which allows to visualize a fluorescent halo made of DNA loops extruded from an insoluble nuclear scaffold, after treating the cells with a nonionic detergent and dehistonized in the presence of a DNA-intercalating dye [7]. They measured intact loops with an average size of 90 kb from mouse cells. They concluded that loops were attached to a skeleton kind of nuclear matrix, appearing as an insoluble, structural framework, and could be unwound by nicking the DNA with DNase I or exposing the samples to UV light. Moreover, they further identified a relationship between DNA loops and replication, as we will discuss later.

In 1982, Buongiorno-Nardelli and colleagues observed with the same technique an average loop size of 90 kb (maximum halo radius of 15 μm) for frog cells [35]. They also plotted the loop size for different species versus the respective replicon sizes, as measured by various groups, and hypothesize a relationship between loop and replicon sizes, which will be further discussed later.

In 1983, Earnshaw and Laemmli developed a method to isolate and deposit intact mitotic chromosomes on electron microscopy grids and measured radial loop sizes of 83 kb ± 29 kb in human metaphase chromosome preparations [36]. They additionally isolated the protein scaffold from where the loops emanated and established their reversible aggregation upon treatment with high levels of Mg++ or NaCl.

After ten years of speculation on the existence of loop organization of DNA, Jackson and colleagues tackled the issue by isolating chromatin from HeLa cells and embedding it in agarose under physiological buffer conditions to avoid any artifact [37]. They could indeed show that some loops may arise as artifacts from nuclei, nucleoids and scaffolds preparation, but they were also able to show that loops ranged from 5 to 200 kb and averaged on a size of 86 kb throughout the cell cycle. Though not observing any size change between mitosis, G_1_ and S-phase, they proposed that loops could still be dynamic structures, which were not detectable by the assay used. They also fitted the data to a standard curve and obtained an average of 118 kb. In a subsequent work [38], they investigated loop sizes further with the physiological lysis method and using electroelution after restriction enzyme DNA digestion. Probing different enzymes and levels of detachment, they could reproducibly observe a size range of 80–90 kb. Moreover, they observed that attachment of loops to the nucleoskeleton was very stable and measured that fragments of about 1 kb remained protected from nuclease attack.

In 2002, Dekker and colleagues developed a technique to unravel the chromatin structure through the frequency of contacts between different genomic sites, by ligation of these sites and following detection by quantitative PCR reactions [9]. This technique of capturing chromosome conformation was subsequently subjected to a variety of improvements and modifications (reviewed in [10]).

In particular, the method was further developed into Hi-C or high conformation capture in 2009 by Lieberman-Aiden *et al*., by combining proximity-based ligation with massively parallel sequencing [39,40]. They applied Hi-C at a 1 Mb resolution to identify ‘megadomains’ of 5–20 Mb, which were further subdivided into 500 kb – 7 Mb sized domains corresponding to the ‘A’ or active compartment, enriched for open chromatin, and the ‘B’ or inactive compartment, enriched for closed chromatin, which together created the plaid pattern in the contacts’ matrices [39].

In 2012, Dixon and colleagues introduced the concept of topologically associated domains (TADs) as largely species- and cell type-conserved megabase-sized domains, which correlated with the constraints of heterochromatic regions and whose boundaries are enriched for the insulator protein CTCF, housekeeping genes, transfer RNAs and SINE retrotransposon elements [41]. They observed highly self-interacting regions at a bin size of less than 100 kb. In mouse embryonic stem cells, they found 2200 TADs with a median size of 880 kb, occupying 91% of the sequenced genome. Most of these TADs were shared across evolution, with more than 50% of genome boundaries that were found in mouse, being present also in humans and vice versa. They showed that TADs were related to, but independent from, previously described organization structures such as the A/B or active/inactive compartments [39], the LAD/non-LAD or lamina-associated/not associated domains [42,43], and the early/late replicating domains [44]. They further reported that CTCF alone is insufficient to determine TADs boundaries, being that the binding of this protein was found enriched at most boundaries but only 15% of its binding sites were located within these boundaries [41].

Two years later, Rao and colleagues achieved a 1 kb-resolved map of the human genome, made of the so-renamed 10000 loop or contact domains [45]. These domains were reported to have a median size of 185 kb (ranging from 40 kb to 3 Mb), to be associated with histone marks and often linking promoters and enhancers, with CTCF sites enriched at the loops’ anchors in a convergent orientation. Furthermore, they identified six compartments with distinct patterns of histone modifications, two of which related to early and mid replicating regions of the previously identified ‘A’ compartment, and the remaining four to be related to facultative or constitutive heterochromatin of the ‘B’ compartment. All boundaries observed were associated with either a sub-compartment transition (occurring circa every 300 kb) or with a loop (occurring circa every 200 kb) and many with both.

In 2017, Schwarzer *et al*. deleted the cohesin-loading factor Nipbl and observed the disappearance of TADs-associated Hi-C peaks but not of A/B compartments [46]. Furthermore, no effect on transcription was detected [46].

Rao *et al*. (2017) similarly reported that cohesin loss eliminated all loop domains while having only minor effect on transcription [47]. The loss of the short-range loops did not affect the histone modification patterns nor the A/B compartments. Moreover, they promoted a fast model of ‘loop-extrusion’ guided by the cooperation between the two architectural proteins CTCF and cohesin, based on the fact that loop domains reform in a few minutes after cohesin recovery [47].

Similarly, Nora *et al*. (2017) observed only minor global transcriptional effects and no change in A/B compartmentalization upon CTCF depletion [48].

In the same year, Wutz and colleagues also showed that cohesin is required for TADs and additionally proved that extended loops were formed once the cohesin release factors Wapl and PDS5 were removed [49]. Hence, CTCF could define the loop boundaries but it would be bypassed if the cohesin unloading factors did not control the length of loops [49].

A recent study by Bintu and colleagues in 2018, applied sequential rounds of FISH after partitioning a target genomic region into 30 kb segments, in order to generate high-resolution spatial maps of chromatin from single cells [50]. They showed that the disappearance of TAD-like structures after cohesin depletion might be in fact an artifact due to averaging at a population level, since single-cell studies revealed that, in the absence of cohesin, the loop boundaries are shifted from cell to cell and, therefore, not detectable as a peek of frequencies at a population level [50].

Still in 2018, using Hi-C methods in combination with imaging methods, Gibcus and colleagues [51] were able to establish using synchronized chicken DT40 cells that, in prophase, consecutive arrays of 60 kb loops are formed followed by, in prometaphase, the formation of 80 kb inner loops nested within 400 kb outer loops in a helical arrangement. They could, furthermore, show that this arrangement is dependent on the condensin family of proteins and that condensins I and II exerted their effects at different levels.

In 2015, Hsieh and colleagues introduced Micro-C, a novel Hi-C method with nucleosome resolution, in which micrococcal nuclease is used instead of restriction enzymes to fragment chromatin [52]. In 2019, Hsieh and colleagues showed by Micro-C that TADs are segregated further into microTADs by the action of transcription factors, cofactors, and chromatin modifiers [53]. Krietenstein and colleagues utilize the same technique to resolve more than 20000 additional looping interactions with single-nucleosome accuracy in comparison to Hi-C [54]. Hansen and colleagues showed by Micro-C that an RNA-binding region in CTCF mediates self-association and that its deletion disrupts half of the CTCF loops, leading to reorganization of TADs [55].

## Insights on chromatin folding through polymer modeling

Since the earliest discoveries on chromatin folding, a variety of models have been proposed. With the advancement of physics, informatics, and machine-learning algorithms, these could be computed in 3D polymer simulations and compared to experimental data.

Already in 1998, Münkel and Langowski [56] simulated human chromosomes by polymer modeling of a fiber arranged into loops and subsequently forming subcompartments. They could indeed reproduce the formation of chromosome territories in interphase cells. The year after, Münkel and colleagues developed the model further by assuming a chromatin fiber folding into 120 kb loops and their arrangement into rosette-like structures [57]. By comparison with experimental data, they found agreement on the overlap, number, and size of subcompartments between the model of chromosome 15 and the observed subchromosomal foci of either early or late replicating chromatin. The model showed also expected distances as observed for specific marker loci using FISH at both the sub- and megabase ranges [57].

In the subsequent years, models describing folding of chromosomes over length scales between 0.5 and 75 Mb based on random loops were proposed by Bohn, Mateos-Langerak and colleagues [58,59]. The model assumed a self-avoiding polymer and defined the probability of two monomers to interact creating a loop and extending through the whole chromosome. They also tested the model using experimental data and were able to obtain chromatin folding within a confined space [59], which agreed with the evidence that chromosomes occupy distinct territories in interphase nucleus [60].

More recently, the strings and binders switch model (SBS) [61] recapitulated well key aspects of chromatin looping, by investigating the interaction between diffusing binders and a free polymer, on which the positions of the binding sites are assigned. These settings allowed investigation of ‘switches’ or conformational changes that the polymer can experience when bound by other proteins. Randomly diffusing binders were shown to be sufficient to dynamically determine TADs, territories, and thermodynamic changes (reviewed in [11]).

Reviving an older concept of loop extrusion dating back to the 1990s (reviewed in [26]) and adding new biochemical evidence, Fudenberg and colleagues proposed in 2016 [25] that chromatin folding into TADs could result from multiple loops being dynamically extruded. This differs from the models where loops are formed by proteins bringing together the ends of a loop. They also proposed that ring-shaped cohesin complexes would be responsible for the extrusion process. Once loaded onto the DNA, cohesin would start extruding a loop until being removed by the releasing factor Wapl or encountering an obstacle. CTCF bound to DNA sites in a convergent orientation would constitute such an obstacle, stalling the loop extrusion and defining boundaries [25].

Nuebler and colleagues [62] more recently proposed that chromosome organization is shaped by both, affinity-driven compartmentalization and loop extrusion processes coexisting, within the cell nucleus in a nonequilibrium state. Active loop extrusion would counteract and compete out the compartmental segregation of active and inactive chromatin while enhancing TADs, affecting only compartments sized between 500 kb and 2 Mb. This nonequilibrium model of loop extrusion could be used to explain compartmental mixing and different experimental findings related to chromatin perturbations, namely removal of either CTCF, cohesin’s loader Nipbl, or its release factor Wapl [62].

In the same year, 2018, Buckle and colleagues speculated that the simple bead-and-spring polymers assume a homogeneous chromatin fiber, which is not reflecting the situation in vivo [63]. Hence, they developed the HiP-HoP or highly predictive heteromorphic polymer model, in which data from epigenetic marks, chromatin accessibility, and CTCF/cohesin anchors were added onto a polymer chain to reproduce the variability of the chromatin fiber along its length [63]. They integrated this heteromorphic chain with diffusing protein bridges and loop extrusion and were able to reproduce the 3D chromatin organization of genomic loci at both population and single-cell level (based on 3 C and FISH data, respectively), being able to describe varying levels of transcriptional activity across cell types [63].

In 2019, polymer simulations by Falk *et al*. [64] based on both Hi-C and microscopy data could highlight the dominating role of heterochromatin (in particular, constitutive heterochromatin) in inducing phase separation, whereas euchromatin interactions were found to be dispensable for compartmentalization. Heterochromatin–heterochromatin interactions lead to the formation of large (micrometer size) compartments and are likely mediated by the affinity between homotypic repetitive elements, modified histones, and heterochromatin associated proteins [64]. In fact, taking constitutive heterochromatin as an example, we could show that increasing the concentration of a single factor (Mecp2) binding DNA by electrostatic as well as modification-specific interactions, resulted in the coalescence of pericentromeric regions into increasingly larger clusters [65]. Accordingly, Solovei and colleagues [66] subsequently demonstrated that, in the absence of attachment to the nuclear periphery in rodent rod cell nuclei, these chromosomal regions completely fuse into a single cluster in the middle of the nucleus.

In 2020, Brackley and Marenduzzo [67] reviewed the string and binders model focusing on the dynamics of multivalent binders, i.e. transcription factors or other proteins, which can bind chromatin at more than one point to form ‘molecular bridges’ that stabilize loops. In the simplest case, interactions can be electrostatic and non-sequence specific and could lead to spontaneous clustering or ‘bridging-induced attraction’, depending on the interaction’s strength or on the protein’s residence time. This can result in a positive feedback in which protein clusters continue to grow and coarsen in a ‘phase separation’ mode. When specific high-affinity binding sites are included in the model, cluster growth is limited due to the looping out of the low affinity (e.g., electrostatic) interaction chromatin stretches. The resulting ‘clouds of loops’ would, in addition, sterically hinder any cluster to merge further, hence stabilizing the microphase separation [67].

To conclude, in living cells, it is highly probable to find a coexistence of different mechanisms variably dictating the chromatin compaction in different subnuclear regions. The various models would contribute differently within each chromatin compartment, with one model being predominant in some compartments but not in others.

## Is genomic function reading the chromatin structure?

**Figure 2.**
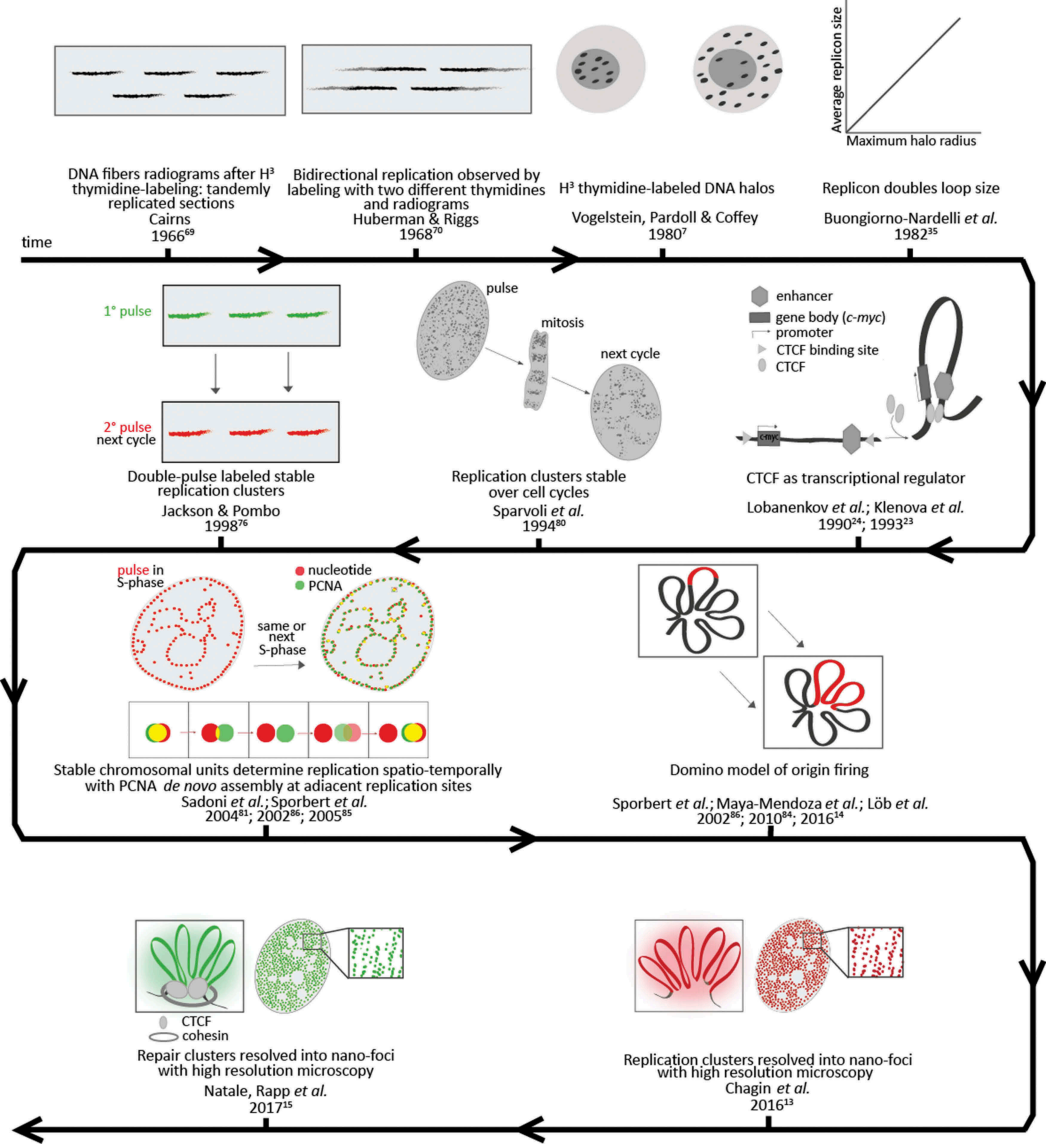
Timeline of measurements and concepts of chromatin functions.

A well-known example of functional chromatin loops is given by the insulation of enhancers and promoters, which are distant from each other on the linear DNA sequence. The looping of the DNA in between the two sites allows these elements to be brought in close proximity and to affect transcription rates. This very interesting escamotage contributed to the fame of CTCF as an insulator protein influencing the transcription of thousands of genes (reviewed in [18]). Transcription might indeed be a function locally defining the chromatin architecture. Although the regulation of transcription can locally define the chromatin structure and vice-versa the chromatin looping can influence the transcription rates, transcription can be very differently regulated depending on the cell state and on the environment and only a small percentage of the whole genome is involved in transcription at any given time. This cell-to-cell variability in relation to the regulation of gene expression is exactly the reason why the influence of the transcriptional function on chromatin looping is so relevant. However, we will focus here on those events of DNA metabolism that are consistent in every cell independently of the cell’s developmental state. In this sense, DNA replication and the repair of DNA damage can be considered as more global events: even though these two processes are also spatio-temporally regulated and, hence, not simultaneously involving the whole genome, they have to cover its full length in a defined time in order to ensure cell proliferation and the correct maintenance of the genome. A selection of studies dealing with replication/repair subnuclear structures is presented below and summarized in Table 2 and timeline Figure 2.

**Table 2.** Sizes of functional chromatin units measured with different methods.

	Reference	Year	Method	Nomenclature/Structure	Organism (cell line)	Median/mean size	Size range
Function	Huberman and Riggs	1968	Labeled DNA autoradiography	Replication sections (IOD)	Hamster and Human	7 – 30 µm (15–60 µm)	(up to 160 µm)
	Lau and Arrighi	1981	Premature chromosome condensation	Replication units	Hamster (CHO)	0.6 µm	0.2–1.2 µm
	Nakamura, Morita and Sato	1986	Conventional microscopy foci analysis	Replication domains	Rat (3Y1-B)	1000 kb	-
	Nakayasu and Berezney	1989	Conventional microscopy foci analysis	Replication granules	Kangaroo (PtK1)	0.5 µm	0.4–0.6 µm (late S up to few µm)
	Jackson and Pombo	1998	Replication labeling on DNA fibers	IOD (eq. to a replicon)	Human (HeLa)	144 kb	25 – 325 kb
			Conventional microscopy foci analysis	Replicon clusters	Human (HeLa)	0.8 Mb	-
	Chagin *et al.*	2016	Replication labeling on DNA fibers	IOD (eq. to a replicon)	Human (HeLa Kyoto)	189 kb	± 121 kb
				IOD (eq. to a replicon)	Mouse (C2C12)	162 kb	± 100 kb
	Natale, Rapp *et al.*	2017	3D-SIM of gH2AX-labeled chromatin	Repair nano-foci	Human (HeLa)	75 kb	34 kb – 159 kb

### Is DNA replication reading the chromatin structure?

Interestingly, already in 1982 Buongiorno-Nardelli and colleagues collected measurements from different studies to propose a relationship between the loop length and the replicon size in different animal and plant species [35]. In DNA halos prepared from radiolabeled frog cells, they observed that radioactivity distributed on a progressively wider area beyond the nuclear matrix at a rate of 0.47 μm/minute. Taking into account replication bidirectionality and the average loop size of 90 kb in frogs, they estimated that one loop would replicate in 30 minutes and, indeed, they did not observe any increase of the labeled area with a pulse of 60 minutes. Similarly, Vogelstein and colleagues had already observed that the radiolabeled DNA moved progressively from the matrix to the halo region, either by increasing the pulse or the chase duration after the pulse [7]. By comparing the loop size estimated with the halo method and the replicon size known from fiber autoradiography studies, Buongiorno-Nardelli and colleagues proposed that the maximum halo radius or loop size is species-specific and that this is directly proportional to the average replicon length in the same species. In fact, they calculated that all species analyzed had an average replicon length four times longer than the maximum halo, which means twice the loop size. Hence, they speculated that a replicon might consist of two adjacent loops, might be read by two matrix-bound replication complexes and have origins and terminations at the anchors of the loops: the newly formed loop would be then released to bind the new matrix [35].

This correlation was possible because other groups had already measured the length of newly synthesized DNA on stretched DNA fibers starting with Cairns in 1963 [68]. From DNA autoradiograms of *E. coli* cultured in H^3^-thymidine, he observed that replication progressed from a fork-like growing point by forming what he called theta-structures (looking like the Greek letter θ). On mammalian DNA fibers, he also showed that the newly replicated DNA appeared as tandemly separated sections [69].

In 1968, Huberman and Riggs confirmed Cairns’ experiments in Chinese hamster and HeLa cells, showing that replication proceeded from an origin in each of the tandemly joined replicating sections [70]. By exploiting thymidines with two different affinities, they observed a bidirectional synthesis progressing in opposite directions from each origin, leading them to propose the bidirectional model of DNA replication. They also proposed the term ‘replication unit’ as the basic unit of replication, meaning that adjacent sections sharing an origin would initiate replication together and hypothesized that replication might proceed until converging with the next growing point.

A variety of subsequent studies [71–73] using nucleotide pulse labeling and microscopical analysis established the existence of functional units of DNA replication in different rodent and marsupial cell lines and, furthermore, described the focal pattern changes throughout S-phase.

Additional analysis of replication labeling performed on stretched DNA fibers determined that the spacing between adjacent origins in mammalian cells varies between 50 and 300 kb (reviewed in [74,75]). This number corresponds to the segment of chromosomal DNA replicated from a single origin of bidirectional DNA replication. This segment is commonly referred to as ‘replicon’.

Jackson and Pombo in 1998, confirmed such numbers and, by analyzing numbers of adjacent replicons in DNA fibers, confirmed that they are activated in clusters [76], as already shown by the earlier fibers studies. Based on pulse-chase labeling of replicating DNA in subsequent cell cycles, these authors proposed that such clusters reflect units of chromosome structure and are stable over cell cycles [76].

In 2010, Guillou and colleagues investigated cohesin’s influence on replication. Cohesin was found enriched at replication origins and found to interact with MCM proteins, as shown by bioinformatics analysis and by immunoprecipitation, respectively [77]. After cohesin depletion, the size of both replicons and DNA loops increased, as shown by DNA combing and DNA halo measurements. In particular, the density of active origins was reduced by three-fold, while the fork speed was maintained, thereby causing a delay in S-phase. Hence, they concluded that cohesin is required for the formation and/or stabilization of loops at replication foci, mediating those long-range interactions which bring together a cluster of origins [77]. The same authors could not observe any delay of replication nor any change in halo size after CTCF depletion, but they speculate this might be due to the transient nature of transcription-related loops [77]. In 2019, Cremer *et al*. analyzed replication nanofoci at high resolution upon cohesin depletion. The nanofoci volume increased, hinting to chromatin relaxation, although the replication patterns were maintained [78]. The fact that loops are dynamic is altogether not incompatible with the hypothesis of a stable structural unit. Loops can dynamically be released and reformed, which gives rise to single-cell variability when taking single snapshots in time [50,79]. However, over time, loops or clusters of loops are stable in the sense that both the focal structures and their replication timing are maintained over multiple cell generations [80,81] (see also below).

Making use of several decades of technological developments, we applied a multi-dimensional approach to perform a comprehensive analysis of replication dynamics in mammalian cells [13,82]. In detail, replication units (as segment of DNA that is synthesized from a single origin by two opposing forks) were extensively analyzed by live cell microscopy of cells stably expressing fluorescent replication factors and by super-resolution microscopy of fixed cells in combination with molecular characterization of replicons in combed DNA fibers and measurement of S-phase duration. In both human and mouse cells, 5000 replication units or foci (RFi) could be counted on average at any sub-stage of S-phase, when imaged at high-resolution by 3D-structured illumination microscopy (3D-SIM) [13]. These data showed that the replication structures commonly observed at conventional resolution light microscopy are not the actual units of replication, but higher-order organization clusters comprising on average 4–5 of the basic units. Our findings on the cluster composition were confirmed with 2D- stochastic optical reconstruction microscopy (STORM) by Xiang and colleagues, which showed that an average cluster consists of four co-replicating regions that are spaced 60 nm apart within a total region of 150 nm [83].

Molecular combing of newly replicated DNA fibers showed that the average replicon size estimated as an inter-origin distance (IOD) was of 188.7 and 161.7 kb, with an average lifetime of 57 and 33 minutes, respectively, in human and mouse cells [13]. The replicon sizes obtained are coincidentally within a two-fold larger size to published loop sizes in mammalian cells (see above, Table 1 and [35–38]).

After measuring the genome size of each cell line used, both, the time to replicate the genome from a single fork as well as the number of replication forks that need to be active in parallel in order to replicate the full genome within the S-phase duration were calculated. This calculated number of required replication forks was divided by two assuming that most replication units are bidirectional. This number was subsequently divided by the actual number of replication nanofoci counted at any given time of S-phase and the result was approximately one (0.92 in human cells) [13]. Bridging these different analyses at different resolutions, it was possible to conclude that most of the replication nanofoci imaged at 3D-SIM represent single (bidirectional) replicons being active in parallel. This indicates that individual replicons could be optically resolved as spatially separated entities, leading to the conclusion that the DNA synthesis machinery should be actually reading structural chromatin units [13].

The folding of chromatin would consequently induce the firing of adjacent origins within the 3D nuclear space, as discussed in our proposed domino-like model of S-phase progression [14,81,84–86]. In more detail, whenever an origin is fired, this would increase the probability of firing of the neighboring origins as in the domino game the fall of one bar would lead the neighboring bars to fall down (see Figure 2). The resolution of chromatin units as replicons thousand times larger than the nucleosomes, in a range of 150–200 kb or bp, respectively [13], led us to propose that these could represent the next level of chromatin organization above the nucleosome level. Furthermore, these chromatin structural units would be read by the DNA replication machinery in a spatio-temporal manner every time the cell needs to duplicate the genome.

### Is DNA damage response reading the chromatin structure?

Another global process that involves the whole genome and might help us to unravel the chromatin organization is the chromatin signaling upon DNA damage (DNA damage response or DDR), which starts with the phosphorylation of the histone variant H2AX (γH2AX) (reviewed in [87,88]). This modification has been proposed to spread up to several Mb from the original site of damage and it can be detected as a focal structure with conventional microscopy. In a recent analysis of 53BP1 focal structures, Kilic and colleagues proposed that also phase separation plays a role in delimiting the DDR [89].

With the use of 3D-SIM and STED (stimulated emission depletion) microscopy, we could show that γH2AX foci are actually clusters of nanofoci with a median DNA size of 75 kb (spanning from 40 to 160 kb) in human cells [15]. The nanofocus DNA content was estimated by applying a novel calculation based on the fraction of genomic DNA in the volume of each singularly segmented nanofocus in relation to the overall DNA content within the full nuclear volume. The measurement of distances between the centroid of all the nanofoci allowed to estimate their clustering. Cluster size distributions had a median DNA size of 921, 623 or 220 kb (ranging from 112 to 938 kb), depending on the time point after irradiation (0.5, 3 or 24 hours post-irradiation, respectively) [15]. The DDR nanofoci are, hence, lower-order units of chromatin organization, which appear to be spatially organized in higher-order clusters within the (sub-)megabase size range. When these foci were imaged together with labeled phospho-Ku70 proteins, as one of the first repair factors known to bind the ends of the double-strand break, *circa* one focus of phospho-Ku70 was present within every cluster of 3–4 γH2AX nano-foci [15]. This indicated that multiple units of γH2AX-decorated chromatin made up a domain in which one single double-strand break was found. Moreover, the signaling of damage and the subsequent DNA repair were both impaired upon depletion of CTCF, which, as mentioned before, is one of the main architectural proteins involved in chromatin looping together with cohesin. In addition, CTCF could also play a role in recruiting repair factors to double-strand breaks [90].

The impairment of DNA repair after CTCF depletion suggested that the DNA damage response is structured by chromatin loops clustered together by CTCF. In particular, after depleting CTCF to 40% of the control protein levels and upon irradiation, γH2AX nanofoci decreased in number, clustering and DNA content [15]. This was consistent with the fact that, in control cells, CTCF was shown to delimit the clusters of γH2AX-decorated chromatin, both through high-resolution single-cell imaging and ChIP-Seq data analysis before and during DDR. According to our findings on the DNA repair nanofoci clustering, each γH2AX nanofocus would be a single loop within a CTCF-delimited multi-loop cluster. Hence, CTCF influences the spreading of the signal of DNA damage through its role in delimiting clusters of repair units. Colony formation assays and measurements of the residual damage through single-cell comet assay demonstrated also that CTCF depletion resulted in radiosensitization and decreased the cellular ability to repair the damaged DNA, supporting its impact on the DNA repair function by its role in chromatin organization [15].

## Is genome structure determining genome function?

It has not escape our notice that replicons have an average size double than that of repair nanofoci, leading us to speculate that a replicon corresponds to two adjacent loops while the DNA damage signaling relies on one single loop [13,15]. This would nicely correlate with the fact that in most species, the replicon has twice the size of a loop, as reported by Buongiorno-Nardelli and colleagues [35]. Additionally, one could further hypothesize that each single loop in the double-loop replicon corresponds to a single fork being part of the bidirectional process of DNA replication.

**Figure 3.**
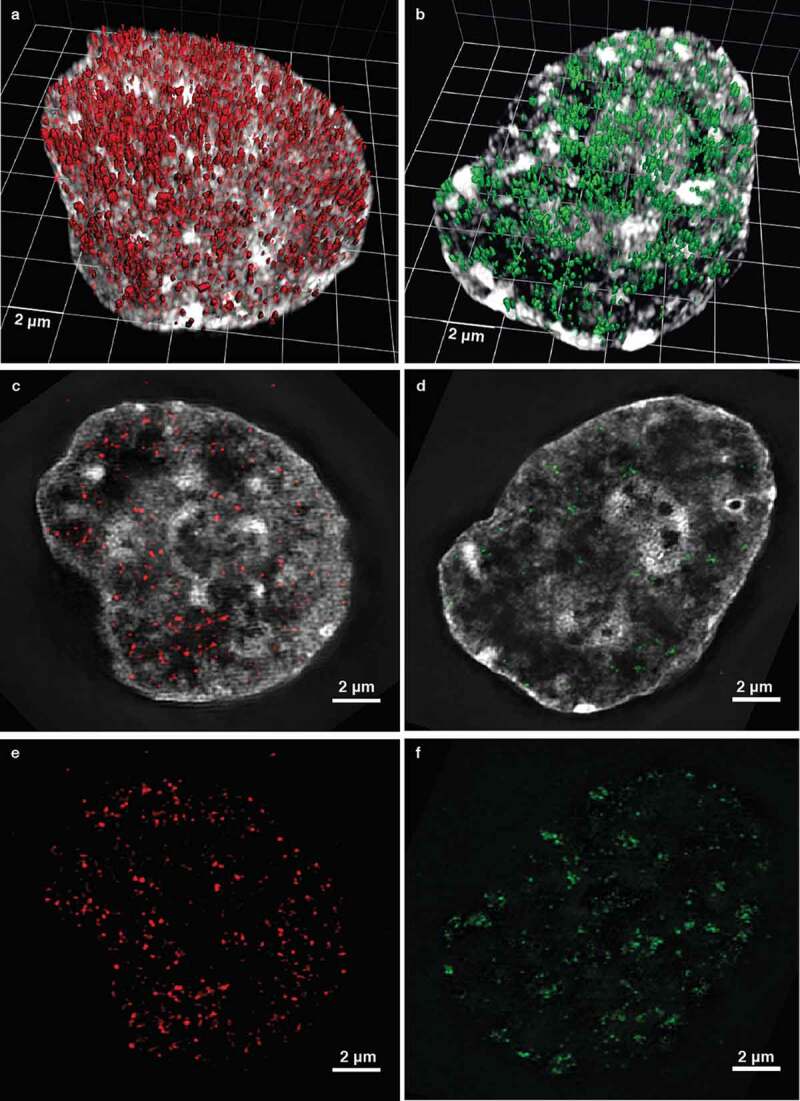
DNA replication and repair units in human HeLa Kyoto cells using 3D-structured illumination microscopy. In (a), is shown a 3D rendering of DNA replication units (red) in a cell labeled during early S-phase by a 10-minute pulse of the thymidine analogue CldU (10 μM) followed by detection using immunostaining. In (b), is shown a 3D rendering of DNA damage response units (green) in a cell irradiated with 5 Gy X-rays, fixed half an hour later, and immunostained for phosphorylated H2AX. Central sections of the same cells as in (a) and (b) are shown in (c) and (d) overlayed with the DNA stained with DAPI and in (e) and (f) without DNA overlay, respectively.

Moreover, in both investigations it was shown that replication and repair foci as seen at conventional microscopy actually consist of clusters of 4–5 nanofoci when observed with super-resolution microscopy, suggesting that the two consisted of multiple loops nested together into a domain [13,15]. Remarkably, the individual replication and damage repair nanofoci are extraordinarily similar at the superresolution light microscopy level and it is, in fact, very difficult to distinguish them when seen side-by-side, as depicted in Figure 3.

Buongiorno-Nardelli and colleagues [35] predicted that replication would faithfully reproduce the chromosome structure at each cell cycle. This can easily be seen by labeling the cells with nucleotides and observing them in live cell microscopy: the replication pattern corresponding to the S-phase stage in which the cells were labeled is stably visible also in subsequent cell cycles, confirming that the structure determining replication units (a.k.a. replicons) is maintained over different generations, as already shown in 1994 by Sparvoli and colleagues in pea root cells using BrdU pulse labeling [80]. Jackson and Pombo also highlighted how individual replicon clusters could be stably detected in HeLa cell nuclei throughout successive cell cycles after BrdU pulse labeling [76]. They made similar observations on stretched DNA fibers, where 95% of replicons labeled in one S-phase could again detected in the next cycle [76].

In 2004, using directly labeled nascent DNA and time-lapse microscopy analysis over subsequent cell cycles we have shown that the replication units are stable sub-chromosomal foci, which are read in a defined temporal and spatial order during DNA synthesis in successive cell cycles [81]. In particular, we found that not only a given replication pattern was maintained through different cell generations, but it also colocalized with the replication machinery during the next phase of DNA synthesis, as detected by simultaneously imaging the Cy3-labeled nucleotides incorporated into newly synthetized DNA and their colocalization with replication machinery components in subsequent cell cycles [81]. Moreover, during the same S-phase, the replication machinery dissociated from one Cy3-labeled focus and reassembled at an adjacent new site. This suggested that the replication machinery reads the sub-chromosomal structures that are spatially next to each other. We further developed this concept into a model of domino-like progression of DNA replication, whereby the replication fork induces the firing of nearby origins [14,81,84–86]. Based on this proximity induced firing and taking into account the 3D folding of chromatin, the model was able to reproduce the spatio-temporal distribution of replication units that is commonly observed during S-phase progression [14].

Another striking similarity is found between the mean size of 185 kb for the so-called ‘contact domains’ measured by Rao and colleagues using Hi-C and the mean inter-origin distance, equivalent to one replicon – 189 kb that we measured on stretched DNA fibers after replication labeling [13,45].

If a replicon, sized as a (double) loop domain (as shown across multiple species and in multiple studies), is made up of two symmetrical forks, each of which has *circa* the same size of a repair nanofocus, we propose that DNA replication and DNA repair, being both global genomic processes, do indeed function by reading a basic chromatin loop unit maintained over cell generations and, hence, genome structure determines its function.

The agreement between the size of a loop, a repair nanofocus, and a replication fork is even more striking when we consider that these measurements were achieved with different techniques. Loop sizes were achieved by DNA halo technique [35], fork sizes were obtained on stretched DNA fibers [13] and repair units by analysis of focal structures *in situ* [15]. Hence, no matter which technique is utilized, the replication and the repair functions rely on the same structural unit, which is a DNA loop of circa 70–90 kb (Table 1). Consequentially, two forks of a bidirectional replicon label a length of DNA that corresponds to a pair of loops, circa 160–190 kb (Table 2). This is also supported by the observations of Buongiorno-Nardelli and colleagues, which showed that, on average, the replicon size is double the loop size in different species [35].

This relationship is further supported by similarities in the kinetics of the two processes. Both replication and repair follow a spatio-temporal order, which is dictated by the fact that euchromatin gets processed earlier than heterochromatin. In both processes, we observe a pan-nuclear pattern of numerous fine foci at earlier stages, whereas focal structures get increasingly clustered at later time points [13,15].

We and others have shown that depletion of cohesin increased loop size and replicon size [77,78] and, in addition, we showed that CTCF brings together single repair nanofoci into a cluster and that its absence impairs the spreading of these nanofoci [15]. As these nanofoci correspond to single loops in size, we hypothesized that CTCF is bringing different loops together in a multi-loop cluster and that this clustering is required for the spreading of the histone modification on the single loops that are brought in proximity. Based on these observations, we can hypothesize that the loops extruded by the cooperation of CTCF and cohesin can have a functional significance in terms of DNA replication and repair. However, future investigations on loop dynamics and the presence and absence of these and other proteins will help us to better elucidate how the structural units of replication and repair are dynamically maintained in living cells.
